# Extracellular Vesicles in Hepatobiliary Malignancies

**DOI:** 10.3389/fimmu.2018.02270

**Published:** 2018-10-12

**Authors:** Ainhoa Lapitz, Ander Arbelaiz, Paula Olaizola, Aitziber Aranburu, Luis Bujanda, Maria J. Perugorria, Jesus M. Banales

**Affiliations:** ^1^Department of Liver and Gastrointestinal Diseases, Biodonostia Research Institute, Donostia University Hospital, University of the Basque Country (UPV/EHU), San Sebastian, Spain; ^2^“Centro de Investigación Biomédica en Red de Enfermedades Hepáticas y Digestivas” (CIBERehd), Carlos III National Institute of Health, Madrid, Spain; ^3^IKERBASQUE, Basque Foundation for Science, Bilbao, Spain

**Keywords:** extracellular vesicles, hepatocellular carcinoma, cholangiocarcinoma, hepatoblastoma, pathogenesis, therapy, diagnosis

## Abstract

Primary hepatobiliary malignancies include a heterogeneous group of cancers with dismal prognosis, among which hepatocellular carcinoma (HCC), cholangiocarcinoma (CCA), and hepatoblastoma (HB) stand out. These tumors mainly arise from the malignant transformation of hepatocytes, cholangiocytes (bile duct epithelial cells) or hepatoblasts (embryonic liver progenitor cells), respectively. Early diagnosis, prognosis prediction and effective therapies are still a utopia for these diseases. Extracellular vesicles (EVs) are small membrane-enclosed spheres secreted by cells and present in biological fluids. They contain multiple types of biomolecules, such as proteins, RNA, DNA, metabolites and lipids, which make them a potential source of biomarkers as well as regulators of human pathobiology. In this review, the role of EVs in the pathogenesis of hepatobiliary cancers and their potential usefulness as disease biomarkers are highlighted. Moreover, the therapeutic value of EV regulation is discussed and future directions on basic and clinical research are indicated.

## Introduction

Liver cancer is a major health problem worldwide, representing the second leading cause of all cancer-related deaths (1). This cancer involves a heterogeneous set of hepatobiliary malignancies including hepatocellular carcinoma (HCC), cholangiocarcinoma (CCA), and hepatoblastoma (HB), which mainly arise from the malignant transformation of hepatocytes, cholangiocytes, and hepatoblasts, respectively (1, 2). Early non-invasive diagnosis, prediction of prognosis and treatment-response, as well as effective personalized therapies are still a challenge, highly compromising patient outcome (3–5).

HCC is the sixth most prevalent malignant tumor (10:100,000 incidence) and is strongly associated (~90%) with the presence of liver cirrhosis (LC) caused by alcohol, viral infections [hepatitis B (HBV) or C (HCV) viruses], and/or steatosis, among others (5, 6). CCA is a rare cancer, but its incidence (~5/100.000) is increasing worldwide. Although the etiology of the majority of CCAs is unknown, several risk factors may predispose for its development, including the presence of primary sclerosing cholangitis (PSC), liver fluke infections (endemic from East Asia), cirrhosis and congenital biliary disorders (3). On the other hand, HB is the most common pediatric liver malignancy, principally affecting children between 6 months and 3 years of age. HB is responsible for up to ~1% of all pediatric cancers, with an annual incidence of 0.5–1.5 cases (4, 7). Despite most HB cases are sporadic, some of them have been associated with hereditary cancer syndromes including familial adenomatous polyposis (FAP) and Beckwith-Widemann syndrome (BWS), as well as with prematurity or low birth weight (4, 7). Since hepatobiliary malignances are usually diagnosed in late stages and are highly chemoresistant, the complete surgical resection of the tumors or liver transplantation constitute the only potential curative options. However, these therapeutic strategies are exclusively indicated under certain strict and conservative clinical criteria (3, 5, 6). Therefore, there is an urgent need to determine new accurate non-invasive biomarkers for the early diagnosis of these diseases, as well as to monitor and predict disease progression and treatment response. Moreover, new effective personalized treatments are desirable in order to improve the outcome and life quality of patients.

During the last decade, extracellular vesicles (EVs) have opened new opportunities for non-invasive diagnosis and monitoring of human diseases. Their presence in biological fluids (serum, urine, bile, saliva, etc.) and their unique and diverse biomolecular composition (proteins, RNA, DNA, metabolites, and lipids) make EVs excellent candidates as a source of biomarkers (8, 9). Furthermore, since EVs participate in intercellular communication in human health and disease, they have been postulated as potential tools or targets for therapy. EVs are small membrane-encapsulated spheres produced and secreted by cells through complex and precise molecular mechanisms (10–13). Traditionally, EVs are classified according to their biogenesis into exosomes, microvesicles (MVs) or microparticles, and apoptotic bodies (11, 12). Exosomes are referred to those EVs produced inside the multivesicular endosomes (MVEs) of the cells. Their morphology is spherical and the size ranges between 40 and 150 nm in diameter (11, 14, 15). Cell MVEs are vesicular entities generated in the maturation process of the early endosomes, and where intraluminal vesicles (ILVs) are formed by the invagination of the MVE membrane. ILVs are the incipient exosomes that are released to the extracellular media upon the fusion of the MVEs with the plasma membrane of the cell (11). On the other hand, MVs or microparticles originate from the direct budding of the cell plasma membrane. Their size (40–1000 nm) and morphology are heterogeneous (15, 16). Apoptotic bodies are vesicles produced by cells undergoing apoptosis. Thus, their size (~40 to 2000–5000 nm) and morphology are diverse (15, 16). Although this classification is widely accepted, to date, there are no specific biomarkers to differentiate exosomes from other types of nano-sized vesicle populations, limiting their specific isolation from biofluids. Therefore, the smallest vesicles (nano-vesicles) present and isolated from biological fluids comprise a mix of exosomes and plasma membrane-derived vesicles.

EVs have changed the paradigm of intercellular communication, which was traditionally restricted to the autocrine, paracrine and endocrine interaction through soluble proteins and lipids, or through direct cell-to-cell contact mediated by proteins, gap junctions, or tunneling nano-tubes in pluricellular organisms (17, 18). Accordingly, EVs contain an aqueous lumen and a specific subset of membrane and soluble proteins, nucleic acids (DNA and RNA), lipids and metabolites that can be horizontally transferred to local or distant cells by direct EV-cell membrane contact, fusion or internalization (12). The importance of EVs is highlighted by the fact that their composition is specific depending on the cell status and on the received stimuli (19, 20), which indicates a certain degree of selective packaging. EVs confer protection to the biomolecules enclosed inside the lipid bilayer, preventing their enzymatic degradation (21).

EVs participate in the regulation of multiple cancer hallmarks. They can transmit oncogenic signals by transferring pro-tumor RNAs and proteins that regulate diverse key processes in tumor progression such as proliferation, survival, differentiation, and invasion/migration of cancer cells (22–30). Additionally, EVs are involved in the crosstalk between tumor cells and stroma, promoting inflammation (31), cell matrix remodeling (32), neovascularization or angiogenesis (33, 34), chemoresistance (35–37), formation of the metastatic niche (38, 39), and inhibition of the anti-tumor immune response (40–42). Therefore, EVs represent key targets for therapy at various levels, including production, release and uptake by target cells. Additionally, the blockage or removal of tumor-derived EVs by apheresis with specific devices constitutes a potential therapeutic approach. Of note, EVs are also excellent candidates for the delivery of new anti-cancer proteins, RNAs, metabolites, drugs or cancer vaccines.

## EV biogenesis and regulation

**Figure 1:**
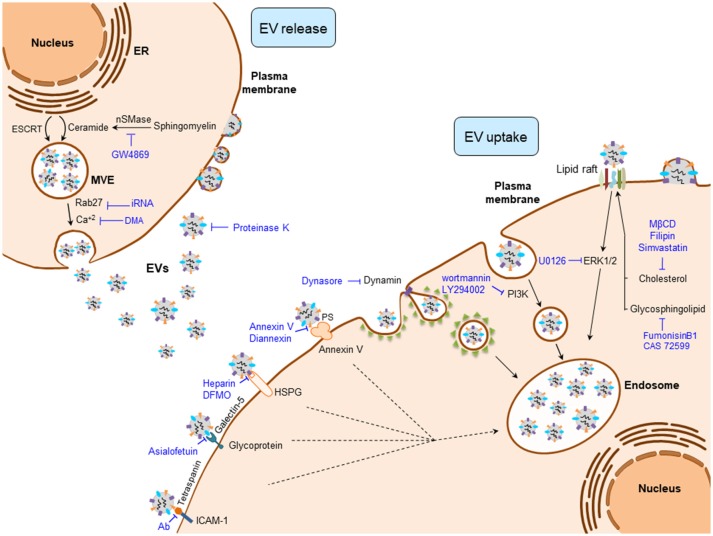
Regulatory mechanisms of EV biogenesis, release, and uptake. Exosome release can be inhibited by interfering their biogenesis (e.g., ceramide production) or the membrane fusion of the multivesicular endosome (MVE) with the plasma membrane (e.g., Rab27). Once EVs are released to the extracellular milieu, their uptake can be blocked by interfering the EV-plasma membrane protein interactions (e.g., Tetraspanins), clathrin- and caveolin-dependent endocytosos (e.g., Dynasore), phagocytosis (e.g., Wortmannin), and by inhibiting lipid-raft mediated endocytosis (e.g., Filipin). DFMO, difluoromethylornithine; DMA, dimethyl amiloride; ESCRT, endosomal sorting complex required for transport; EVs, extracellular vesicles; HSPG, heparan sulfate proteoglycans; ICAM-1, intercellular adhesion molecule 1; MβCD, methyl-β-cyclodextrin; nSMase, neutral sphingomyelinase; PS, phosphatidylserine.

EV production is a highly regulated and complex cellular process where several protein networks and diverse intracellular signals are involved (Figure 1). Among the different EV populations, the exosome production machinery is the best studied. Nevertheless, both exosomes and MVs share numerous mechanisms that participate in their biogenesis, release, and uptake (43). Regarding the mechanisms involved in the biogenesis of exosomes, the endosomal sorting complex required for transport (ESCRT) machinery has been reported to participate in MVE and ILV generation (44). However, ESCRT independent mechanisms have also been described in the formation of exosomes, including ceramide production by neutral type II sphingomyelinase (nSMase2) (45, 46), lipid rafts (47), phospholipase D2 (PLD2) (48, 49), and tetraspanin family of proteins (e.g., CD9, CD63, and CD81) (50, 51), which form dynamic membrane microdomains that promote their budding and assure exosome formation.

Intracellular trafficking of MVEs is coordinated by the cytoskeleton and motor proteins such as dynein (52) and Rab family of GTPases (Figure 1) (53). MVEs fuse with the plasma membrane via SNARE proteins, finally allowing exosome secretion (53, 54). In contrast, MVs arise as a result of the direct budding of the cell plasma membrane. Their biogenesis requires Ca^2+^-dependent membrane phospholipid and cytoskeleton rearrangements, which enable MV blebbing and release (55). Released EVs are recognized by the recipient cells through specific interactions between their membrane components. These include integrins, lipids, tetraspanins, proteoglycans, among others (56). Plasma membrane-bound EVs can be internalized through clathrin-mediated endocytosis (CME) or clathrin-independent processes that include phagocytosis, macropinocytosis and lipid rafts.

Several experimental strategies have targeted the aforementioned mechanisms to interfere with EV production, release and uptake at different levels (Figure 1). Among these, intervention on ceramide production has been the most widely used strategy to decrease exosome production in cancer cells and thereby abolish the multiple oncogenic effects of tumor-derived exosomes in several cancers. Experimental inhibition of nSMase2, responsible for ceramide production, with its inhibitor GW4869 reduces exosome secretion (45, 57) and sensitizes cancer cells to chemotherapy (58). Of note, the presence of GW4869 inhibits the migratory capacity of CCA cells (31). Several intercellular signals involved in the regulation of the EV production are also under investigation, including the reduction of intracellular Ca^2+^ concentration. In fact, the Na^+^/Ca^+2^ exchange inhibitor dimethyl amiloride (DMA) leads to diminished EV production in lymphoma cells, resulting in an enhanced anti-tumor immune response (40). Regarding the proteins that participate in the transport of exosomes, Rab family proteins are key mediators of MVE transit to the plasma membrane, their inhibition being linked to a decrease of exosome release (59). Accordingly, the repression of Rab27a diminished growth and dissemination of cancer cells *in vivo* (39, 60).

Different complex mechanisms, including protein and/or lipid interactions between EV and recipient cell surface components, are required for the EV cell uptake (Figure 1). These mechanisms include phagocytosis, macropinocytosis, clathrin, and caveolin dependent endocytic pathways, as well as lipid raft-mediated and membrane fusion processes (56). Therefore, aiming to block tumor EV uptake processes, different components of these machineries have been targeted. Membrane proteins including tetraspanins, integrins, lectins, proteoglycans, major histocompatibility complex (MHC) molecules, glycoproteins and other receptors are involved in EV-recipient cell interaction. Tetraspanins, enriched proteins present in EVs and well-established markers of these vesicles (61, 62), participate in EV-cell surface adhesion mediating their uptake. Thus, antibody-based inhibition of the CD81 and CD9 tetraspanins as well as the blockade of αV and β3 integrins, hampers EV uptake (63, 64). Lectins, such as galectin-5, can also be targeted with the glycoprotein asialofetuin to interfere with EV-cell interaction and the subsequent cellular internalization (65). Likewise, targeting the lectin receptors DC-SIGN or DEC-205 with specific antibodies also results in a reduction of EV uptake (66, 67). Heparin can also block the internalization of cancer EVs by binding to the cell surface heparan sulfate proteoglycans (68, 69). Furthermore, the pivotal interaction between EVs and the plasma membrane of the cell can be partially inhibited by proteinase K treatment, blocking EV recognition and the subsequent endocytic process in cancer cells (70).

The best studied EV internalization mechanisms are related with the endocytic pathway (63, 70, 71). Since these processes depend on the cytoskeleton, the inhibition of actin polymerization by cytochalasin D reduces EV uptake by phagocytosis (56, 70, 71). In addition, the uptake of EVs by macrophages can be abrogated by the inhibition of phosphoinositide-3-kinase (PI3K) with wortmannin or LY294002 (72). Moreover, inhibitors of macropinocytosis or CME [5-ethyl-N-isopropyl amiloride (EIPA) and chlorpromazine, respectively] reduce tumor-derived EV internalization (70). Dynasore can also impair CME through the inhibition of dynamin 2, needed for clathrin-coated endosome membrane fission (65, 73–76). On the other hand, certain endocytic processes are closely related to lipid rafts, and the intervention on their composition impairs the uptake of EVs. Thus, the use of the glycosphingolipid synthesis inhibitors [i.e., fumosinin B1 and N-butyldeoxynojirimycin hydrochloride (also known as CAS72599)] reduces EV uptake (77). Cholesterol reducing agents including methyl-beta-cyclodextrin (MβCD) (70, 78, 79), filipin (71, 79) and simvastatin, as well as the inhibition of ERK1/2 signaling by U0126 may also impair the uptake of EVs (79). Masking phosphatidylserine (PS), present in the membrane surface of EVs, by Diannexin and blocking its receptor TIM4 inhibits epidermal growth factor receptor (EGFR) transfer from tumor EVs to endothelial cells resulting in reduced tumor growth and microvascular density *in vivo* (80). EVs can also release their content into the recipient cells by the direct fusion of plasma and EV membranes. This fusion is enhanced in acidic conditions, a general feature of cancer cells (81). In this sense, proton pump inhibition leads to reduced EV uptake by cancer cells (82).

## EVs in hepatobiliary cancers

### Hepatocellular carcinoma

HCC cell-derived EVs participate in autocrine and/or paracrine cellular communications, regulating tumor growth, chemoresistance, angiogenesis, and dissemination. Several lines of evidence indicate that HCC cell-derived EVs promote tumor resistance against chemotherapeutic drugs such as sorafenib, doxorubicin or camptothecin. For instance, an enrichment of long intergenic non-coding RNA regulator of reprogramming (linc-ROR) in EVs derived from sorafenib-treated HCC cells prevents chemotherapy-induced apoptosis through p53 repression and increases the expression of tumor-initiating liver cancer stem cell CD133 marker (83). Another molecular mechanism involved in HCC cell-derived EV-induced sorafenib resistance includes the activation of the hepatocyte growth factor (HGF)/c-Met/Akt signaling pathway in liver cancer cells (84).

EVs derived from HCC cells may also regulate angiogenesis (85). Experimental *in vitro* models indicate that EVs derived from CD90+ liver cancer cells (i.e., cancer stem-like cells present in primary tumors and blood of HCC patients, associated with metastasis as well as bad prognosis) are enriched in long non-coding RNA (lncRNA) H19, which promotes the expression of vascular endothelial growth factor (VEGF) and its receptor VEGF-R1 in endothelial cells. Moreover, lncRNA H19 stimulates tube formation as well as cell-adhesion properties in endothelial cells, inducing the expression of intercellular adhesion molecule 1 (ICAM-1) in this cell-type.

Concerning the role of EVs in metastasis, several studies have reported that EVs secreted from HCC cells or from adjacent cells are also involved in the promotion of tumor cell metastasis (86). Transcriptomic and proteomic profiling revealed that EVs derived from metastatic HCC cells carry a larger number of pro-tumorigenic RNAs and proteins, such as MET proto-oncogene, S100 family members (S100A4, S100A10, and S100A11) and the caveolins (CAV1 and CAV2). HCC-derived EVs trigger the activation of PI3K/Akt and MAPK signaling pathways and the secretion of active MMP2 and MMP9 matrix metalloproteinases (MMPs) in hepatocytes, which in turn enhance their migratory and invasive ability. On the other hand, cancer-associated fibroblast (CAF)-derived EVs may also contribute to HCC cell proliferation and metastasis (87). Thus, a reduction in the miR-320a level was observed in CAF-derived EVs compared to para-cancer fibroblasts (PAFs). This miR-320a directly targets pre-B-cell leukemia transcription factor 3 (PBX3), suppressing HCC cell proliferation, migration, and invasion. The anti-tumor effects of miR-320a were confirmed *in vivo* using HCC tumor xenograft models, in which tumor growth was inhibited when HCC cells were co-injected with miR-320a over-expressing CAFs into nude mice. Besides EVs derived from CAFs, innate immune cell-derived MVs have also been reported to enhance HCC metastasis through CD11b and CD18, also known as integrin α_M_β_2_ (88).

### Cholangiocarcinoma

The presence of EVs in bile and their role regulating cholangiocyte physiology was first described in murine models (89). However, EVs also play a role in biliary pathobiology. In CCA tumors, several reports have emphasized the importance of EVs in the regulation of the interplay between CCA cells and the cells present in the tumor stroma. CCA cell-derived EVs favor the fibroblastic differentiation of bone marrow-derived mesenchymal stem cells (MSCs) and the secretion of pro-inflammatory cytokines and chemokines, including interleukin (IL)-6, chemokine (C-X-C motif) ligand 1 (CLXC1), and chemokine (C-C motif) ligand 2 (CCL2/MCP-1), which ultimately stimulate CCA cell proliferation via IL6/STAT3 signaling pathway (31).

CCA-derived EVs may contain oncogenic biomolecules not only involved in modulating inflammatory and proliferative responses but also controlling migratory and metastatic processes. Two studies employing comparative proteomic approaches have explored the protein content of CCA-derived and cholangiocyte-derived EVs *in vitro*, identifying significant differences and a particular oncogenic protein profile related to proliferation and motility in cancer cell-derived EVs (90, 91). Differentially expressed proteins involved in cholangiocarcinogenesis included EGFR, Mucin-1, integrin β4 (ITGB4), and epithelial cell adhesion molecule (EPCAM) (90). EGFR participates in CCA progression, favoring the dedifferentiation and invasiveness of tumor cells and represents a bad prognostic factor (92, 93). Similarly, Mucin-1 and EPCAM, which are also upregulated in CCA, correlate with poor outcome in patients with CCA (94–96). Interestingly, ITGB4 has recently been described as an EV integrin that dictates future metastatic sites, contributing to preferential organotropism of tumor cells (38). On the other hand, EVs secreted by liver-fluke associated CCA cells induce cholangiocyte proliferation (97) and invasion (91), events that are associated with an enrichment of oncoproteins in EVs, including galectin-3 binding protein (LG3BP), prostaglandin F2 receptor negative regulator, 4F2 cell-surface antigen heavy chain (4F2hc), integrin-β1 and EPCAM (91).

## Non-invasive biomarkers

The presence of EVs in biological fluids and their diverse molecular cargo has recently placed EVs as a new source of non-invasive disease biomarkers. Indeed, potential biomarker candidates (miRNAs and proteins) have been described in serum- and bile-derived EVs for the diagnosis and/or the prognosis prediction of HB, HCC, and CCA (Table 1).

**Table 1:** EVs as non-invasive biomarkers of hepatobiliary malignancies.

**Disease**	**Name**	**Biomarker type**	**EV source**	**Number of patients**	**Expression**	**SEN (%)**	**SPE (%)**	**AUC**	**References**
HB	miR-21	miRNA	Serum	HB (*n* = 32) vs. Healthy individuals (*n* = 32)	Up	—	—	0.861	(98)
	miR-34a^*^		Serum	HB (*n* = 63) vs. Healthy individuals (*n* = 63)	Down	—	—	0.963	(99)
	miR-34b^*^					—	—		
	miR-34c^*^					—	—		
HCC	LG3BP	Protein	Serum	HCC (*n* = 29) vs. Healthy individuals (*n* = 32)	Up	96.6	71.8	0.904	(90)
	PIGR					82.8	71.8	0.837	
	A2MG					92.9	56.2	0.796	
	MV (ug/mL)	Microvesicle concentration	Blood	Stage I HCC (*n* = 28) vs. Cirrhosis (*n* = 40)	Up	—	—	0.83	(100)
				Stage II HCC (*n* = 20) vs. Cirrhosis (*n* = 40)	Up	—	—	0.94	
	AnnexinV^+^ EpCAM^+^ (microparticle/mL)	TAMP concentration	Serum	HCC (*n* = 86) vs. Healthy individuals (*n* = 58)	Up	—	—	0.77	(101)
	AnnexinV^+^ EpCAM^+^ ASGPR1^+^ (microparticle/mL)		Serum	HCC (*n* = 86) vs. Cirrhosis (*n* = 49)	Up	—	—	0.73	
CCA	FIBG	Protein	Serum	iCCA (*n* = 12) vs. HCC (*n* = 29)	Up	83.3	89.6	0.894	(90)
	A1AG1					83.3	82.1	0.845	
	VTDB					75	89.2	0.823	
	AMPN		Serum	CCA (*n* = 43) vs. Healthy individuals (*n* = 32)	Up	90.7	65.6	0.878	
	VNN1					72.1	87.5	0.876	
	PIGR					83.7	71.8	0.844	
	PIGR		Serum	CCA I-II (*n* = 13) vs. Healthy individuals (*n* = 22)	Up	75	95.4	0.905	
	AMPN					91.7	72.7	0.833	
	FIBG					100	68.1	0.833	
	FIBG		Serum	CCA (*n* = 43) vs. PSC (*n* = 30)	Up	88.4	63.3	0.796	
	A1AG1					76.7	70	0.794	
	S10A8					69.8	66.6	0.759	
	FCN2		Serum	CCA I-II (*n* = 13) vs. PSC (*n* = 30)	Up	100	80.9	0.956	
	ITIH4					91.7	80.9	0.881	
	FIBG					91.7	80.9	0.881	
	miR-191^*^	miRNA	Bile	CCA (*n* = 46) vs. Control (*n* = 50; including PSC, biliary obstruction and bile leak)	Up	67	96	—	(102)
	miR-486-3p^*^								
	miR-1274b^*^								
	miR-16^*^								
	miR-484^*^								
	ENST00000588480.1^*^	lncRNA	Bile	CCA (*n* = 35) vs. Control (*n* = 56)	Up	82.9	58.9	0.709	(103)
	ENST00000517758.1^*^								
	Nanoparticles/L	EV concentration	Bile	Malignant CBD stenoses (pancreatic cancer; *n* = 10 and CCA; *n* = 5) vs. nonmalignant CBD stenoses (chronic pancreatitis; *n* = 15)	Up	—	—	1	(104)
	AnnexinV^+^ EpCAM^+^ ASGPR1^+^ (microparticle/mL)	TAMP concentration	Serum	CCA (*n* = 38) vs. Cirrhosis (*n* = 49)	Up	—	—	0.63	(101)
Liver cancer (HCC/CCA)	AnnexinV^+^ EpCAM^+^ ASGPR1^+^ (microparticle/mL)	TAMP concentration	Serum	Liver tumor (HCC; *n* = 86 and CCA; *n* = 38) vs. Cirrhosis (*n* = 49)	Up	—	—	0.7	(101)

*A1AG1, alpha-1-acid glycoprotein 1; A2MG, alpha-2-macroglobulin; AMPN, aminopeptidase N; ASPGPR1, asialoglycoprotein receptor 1; AUC, area under the receiver operating curve; CBD, common bile duct; CCA, cholangiocarcinoma; EpCAM, epithelial cell adhesion molecule; FCN2, ficolin-2; FIBG, fibrinogen gamma chain; iCCA, intrahepatic cholangiocarcinoma; ITIH4, inter-alpha-trypsin inhibitor heavy chain H4; HB, hepatoblastoma; HCC, hepatocarcinoma; LG3BP, galectin-3-binding protein; lncRNA, long non-coding RNA; miR, microRNA; MV, microvesicle; PIGR, polymeric immunoglobulin receptor; PSC, primary sclerosing cholangitis; SEN, sensitivity; SPE, specificity; TAMP, tumor-associated microparticle; VNN1, pantetheinase; VTDB, vitamin-D binding protein*.

* *biomarker panel*.

In HB patients, serum EV miR-21 levels were higher than in healthy children, and negatively correlated with patient survival (98). On the other hand, decreased levels of miR-34a, miR-34b, and miR-34c were reported in serum EVs from HB infants compared to healthy individuals. Combination of these miRs showed higher diagnostic value than the gold standard alpha fetoprotein (AFP) (99). Furthermore, reduced levels of the miR-34 panel in EVs of HB were associated with lower overall survival (99).

In HCC patients, levels of miRs 18a, 221, 222, and 224 in serum EVs were found upregulated compared to patients with chronic hepatitis B (CHB) or liver cirrhosis, patients, whereas miR-101 level was downregulated (105). Likewise, increased expression of miR-21 was identified in serum EVs from patients with HCC compared to CHB patients or healthy individuals, and correlated with cirrhosis and advanced tumor stage (106). MiR-665 in serum EVs may also be a potential prognostic biomarker for HCC, as high miR-665 levels positively correlated with larger tumor size, local invasion and advanced clinical stages (stage III/IV), and negatively with overall survival (107). Moreover, diminished levels of several miRNAs in serum EVs have been suggested as predictors of HCC recurrence or overall survival (108, 109). MiR expression profiling in serum EVs identified the tumor suppressor miR-718 downregulated in patients with larger tumor diameters and recurrence. Reduced miR-718 expression also correlated with poor histological tumor cell differentiation (108). Furthermore, low levels of miR-125b in serum EVs have been linked to advanced TNM stages and encapsulation, suggesting this miR as a potential prognostic candidate of recurrence and overall survival (109). Besides miRNAs, different proteins present in serum EVs such as LG3BP, polymeric immunoglobulin receptor (PIGR) and alpha-2-macroglobulin (A2MG) were found upregulated in HCC patients compared to healthy individuals, with a better diagnostic value than AFP (90). Apart from changes in the EV cargo, the EV concentration itself could also serve as a disease biomarker. In fact, stage I and II HCC patients showed higher EV concentration in serum compared to patients with liver cirrhosis (100).

In CCA, a panel of miRs (191, 486-3p, 1274b, 16, 484) was found upregulated in bile EVs of patients with CCA compared to a control group containing PSC, biliary obstruction and bile leak syndrome patients (102). The analysis of the lncRNA profile in bile EVs from CCA patients vs. patients with biliary obstruction identified the upregulation of two lncRNAs (i.e., ENST00000588480.1 and ENST00000517758) in CCA patients (103). The combined expression of both lncRNAs showed relevant diagnostic and prognostic value, being increased in advanced TNM stages (III-IV) and showing worse overall survival at high lncRNA concentrations. On the other hand, different proteins present in serum EVs exhibited high diagnostic values when comparing CCA patients with healthy individuals, such as aminopeptidase N (AMPN), pantetheinase (VNN1), and PIGR (90). Some proteins present in serum EVs, such as ficolin-2 (FCN2), inter-alpha-trypsin inhibitor heavy chain H4 (ITIH4) and fibrinogen gamma chain (FIBG), displayed better diagnostic values than CA19-9 (a non-specific tumor marker for the diagnosis of CCA) in the differential diagnosis between CCA (stage I-II) and PSC (90). Nowadays, the differential diagnosis between intrahepatic CCA (iCCA) and HCC by non-invasive methods is not feasible and compromises adequate treatment. In this regard, proteins present in serum EVs—such as FIBG, alpha-1-acid glycoprotein 1 (A1AG1) and vitamin-D binding protein (VTDB)—exhibited higher accuracy than CA19-9 and AFP for the differential diagnosis of iCCA vs. HCC (90). As aforementioned, the EV concentration analysis could also be relevant for the diagnosis of malignant biliary diseases. In this regard, bile EV concentration was reported to accurately discriminate between malignant common bile duct (CBD) stenosis and nonmalignant CBD stenosis (104). In addition, elevated concentration of AnnexinV/EpCAM/ASGPR1 positive tumor-associated microparticles (TAMPs) allowed the diagnosis of patients with liver cancer (HCC and CCA) compared to cirrhotic patients, while no changes were detected between HCC and CCA (101). Notably, the levels of these TAMPs decreased 7 days after the surgical resection of liver tumors, closely relating this microparticle population with tumor presence.

## Therapeutic implications

The use of EVs in anti-cancer therapy is currently under investigation. As EVs carry different types of molecules, they can be used as vehicles to deliver therapeutic cargo into cancer cells (110). Moreover, EVs have shown the ability to modulate the immune system, and to stimulate the immune response against tumor cells (111).

### Molecule carriers

EVs as therapeutic delivery systems provide benefits for the carried therapeutic molecule. Hence, encapsulation of therapeutic compounds (such as chemicals, RNAs, DNAs, proteins, or lipids) increases their bioavailability by preserving their integrity and biological activity, as well as protecting them from enzymatic degradation in biological fluids (112). In comparison to other therapeutic vectors such as synthetic nano-particles, liposomes or recombinant viral vectors, EVs are generally non-immunogenic in nature, which enhances their resistance to fast clearance from circulation (112). EVs also display low toxicity and are quite stable in tissues and circulation, representing adequate therapeutic delivery systems against cancer (113). Furthermore, cell type-specific proteins within EVs seem to provide certain cell tropism (112).

The strategy of using EVs as therapeutic molecule delivery vehicles is starting in liver cancer, mainly focusing on miRNAs. Stellate cell-derived EVs loaded with miR-335-5p, a tumor suppressor miR downregulated in HCC, inhibits HCC cell invasiveness *in vitro* and induces HCC tumor shrinkage *in vivo* through the repression of proliferation and stimulation of apoptosis (114). Moreover, miR-122 enriched EVs obtained from adipose tissue-derived mesenchymal stem cells (ADMSCs) increases HCC cell sensitivity to the chemotherapeutic agents sorafenib and 5-FU (115). The underlying mechanism regulating chemosensitivity consists on the downregulation of miR-122 target genes including cyclin G1 (CCNG1), disintegrin and metalloproteinase domain-containing protein 10 (ADAM10), and insulin-like growth factor 1 receptor (IGF1R), which induce apoptosis and cell cycle arrest *in vitro*. Furthermore, intra-tumor injection of miR-122-enriched EVs in a HCC xenograft mouse model synergized the inhibitory effect of sorafenib *in vivo*, reducing tumor size (115).

In CCA, stellate cell-derived EVs carrying miR-195 inhibited CCA growth and invasiveness *in vitro* (116). Tail vein injection of miR-195 loaded EVs into an orthotopic rat model of CCA reduced tumor size and improved the overall animal survival (116). These anti-neoplasic effects are likely mediated via targeting VEGF, cell division control (CDC) proteins 25 and 42, as well as cyclin-dependent kinases (CDK) 1, 4, and 6.

### Immunotherapy

An alternative therapeutic strategy contemplates the use of EVs as stimulators of the immune system in order to elicit a nontoxic, systemic, and long-lived anti-tumor immune response. Different studies have described the potential use of EVs as immunostimulatory entities against HCC (117–121). For instance, HCC cells under stress conditions, such as heat shock or chemotherapeutic anti-cancer drug treatment, increased EV secretion and surface expression of heat shock proteins (HSPs) (117). HSP-bearing EVs can boost natural killer (NK) cell-mediated cytotoxic response against HCC cells *in vitro* (117). Similarly, histone deacetylase inhibitor MS-275 enhanced the protein levels of immunostimulatory molecules [MHC class I polypeptide-related sequence B (MICB) and HSP70] in EVs derived from HCC cells, increasing the cytotoxicity of NK cells and anti-tumor response (118). The anti-HCC tumor immune response can also be induced by ADMSC-derived EVs, which promote natural killer T cell (NKT) anti-tumor response, thereby facilitating HCC suppression (119).

Alternatively, HCC cell-derived EVs display HCC antigens AFP and glypican 3. Capture of these EVs by dendritic cells (DCs) triggers a strong DC-mediated T cell dependent anti-tumor immune response both *in vitro* and in ectopic and orthotopic *in vivo* mouse models (120). EVs from antigen presenting cells (APCs) can also induce anti-tumor immune responses against HCC. EVs derived from AFP-expressing DCs are able to trigger potent antigen-specific anti-tumor immune responses and reshape the tumor microenvironment from an immunoinhibitory to an immunostimulatory setting in diverse HCC mice models including ectopic, orthotopic and carcinogen-induced HCC (121). Thus, AFP-expressing DC-derived EVs stimulate antigen-specific anti-tumor immune responses *in vivo*, eliciting suppression of tumor growth and prolonging mice survival (121).

## Concluding remarks and future directions

Early diagnosis and treatment of hepatobiliary malignancies is still far from being manageable. The development of non-invasive diagnostic and disease monitoring tools represents a major challenge. The presence of EVs in biological fluids, as well as their capacity to carry tumor-associated molecules, make EVs excellent candidates for clinical application. Hereof, certain progress is being made in the potential use of EVs as a source of non-invasive disease biomarkers. EV concentration as well as their specific cargo can serve as indicators of the different pathological stages of a disease, including the discrimination between early and late phases, and estimation of recurrence and metastasis risk. For that matter, the application of *omic* technologies has provided some potential candidate biomarkers. However, in order to transfer knowledge into the clinical practice, several limitations, and concerns should be considered: (i) different EV isolation procedures (i.e., ultracentrifugation, size exclusion, immune-affinity isolation, polymeric precipitation, and microfluidics) are currently used, providing diverse EV populations and yield depending on the nature of the isolation protocol (ii) a proper characterization of the EVs fraction should be performed. There are minimal experimental requirements defined by the International Society for Extracellular Vesicles (ISEV) (122), which include the analysis of the EV quantity [e.g., nanoparticle tracking analysis (NTA), IZON qNano technique, flow cytometry], size [e.g., NTA, IZON qNano technique, electron microscopy, dynamic light scattering (DLS)], and presence of specific surface markers (e.g., immunoblot, immune-gold electron microscopy) (122, 123), (iii) specific EV markers to distinguish EV subpopulations according to their origin (e.g., exosomes, MVs, apoptotic bodies) are still missing (122), (iv) appropriate clinically-relevant control groups with biopsy-proven diagnosis, as well as a representative number of samples should be included to ensure the accuracy (sensitivity, specificity, AUC, predictive and likelihood ratio values) and significance of the results (124), (v) candidate biomarkers identified in a discovery phase must be internationally validated using easily transferable methodologies into the clinical settings (e.g., ELISA, qPCR), ideally using raw biological fluids (i.e., serum, urine, saliva) and avoiding the costly and time consuming EV isolation techniques.

EVs represent a new opportunity for cancer therapy. They participate in the development and progression of cancer, including the formation of a pro-tumorigenic microenvironment, angiogenesis, chemoresistance, and the generation of a metastatic niche, promoting tumor growth, and aggressiveness. Therefore, interfering the EV biogenesis and/or release may be a potential therapeutic strategy. Several inhibitors targeting these crucial steps have been developed (Figure 1), although their safety and efficacy should be clinically evaluated in the future. Nevertheless, additional regulatory mechanisms of EV generation (e.g., loading), trafficking and autocrine/paracrine signal transduction (e.g., recipient cell internalization routes of specific EV subpopulations) need to be elucidated, which could provide other targets for therapy (125). On the other hand, EVs could be used as drug delivery systems and as immunomodulators promoting anti-tumor response. For drug delivery, a major challenge represents the specific cell targeting *in vivo*, as well as the use of immunologically inert and biocompatible EVs. In contrast, the capacity of EVs to regulate the immune system opens new opportunities for targeting malignancies and for developing anti-tumor vaccines (126).

In conclusion, EVs represent an emerging and stimulating field of research in liver cancer with multiple potential applications, from biomarker discovery to therapy. Nonetheless, thorough research is still needed to gain knowledge on their intrinsic role in liver health and disease, and to evaluate their potential clinical application.

## Author contributions

All authors listed have made a substantial, direct and intellectual contribution to the work, and approved it for publication.

### Conflict of interest statement

The authors declare that the research was conducted in the absence of any commercial or financial relationships that could be construed as a potential conflict of interest.

## Abbreviations

4F2hc: 4F2 cell-surface antigen heavy chain
A1AG1: alpha-1-acid glycoprotein 1
A2MG: alpha-2-macroglobulin
ADAM10: disintegrin and metalloproteinase domain-containing protein 10
ADMSCs: adipose tissue-derived mesenchymal stem cells
AFP: alpha-fetoprotein
AMPN: aminopeptidase N
ASGPR1: asialoglycoprotein receptor
APCs: antigen presenting cells
ASPGPR1: asialoglycoprotein receptor 1
AUC: area under the receiver operating curve
BWS: Beckwith-Widemann syndrome
CAF: cancer associated fibroblast
CBD: common bile duct
CCA: cholangiocarcinoma
CCL2/MCP-1: chemokine (C-C motif) ligand 2
CCNG1: cyclin G1
CDC: cell division protein
CDK: cyclin-dependent kinase
CHB: chronic hepatitis B
CME: clathrin-mediated endocytosis
CXCL1: chemokine (C-X-C motif) ligand 1
DC: dendritic cell
DMA: dimethyl amiloride
EGFR: epidermal growth factor receptor
EIPA: 5-ethyl-N-isopropyl amiloride
EPCAM: epithelial cell adhesion molecule
ESCRT: endosomal sorting complex required for transport
EVs: extracellular vesicles
FAP: familial adenomatous polyposis
FCN2: ficolin-2
FIBG: fibrinogen gamma chain
HB: hepatoblastoma
HBV: hepatitis B virus
HCV: hepatitis C virus
HCC: hepatocellular carcinoma
HGF: hepatocyte growth factor
HSPs: heat shock proteins
HSPG: heparin sulfate proteoglicans
ICAM-1: intercellular adhesion molecule 1
IGF1R: insulin-like growth factor 1 receptor
IL: interleukin
ILVs: intraluminal vesicles
ITGB4: integrin β4
ITIH4: inter-alpha-trypsin inhibitor heavy chain H4
LC: liver cirrhosis
LG3BP: galectin-3-binding protein
linc-ROR: long intergenic non-protein coding RNA regulator of reprogramming
lncRNA: long non-coding RNA
MβCD: methyl-b-cyclodextrin
MHC: major histocompatibility complex
MICB: MHC class I polypeptide-related sequence B
MMP: matrix metalloproteinase
MSC: mesemchymal stem cell
MVs: microvesicles
MVE: multivesicular endosome
NK cell: natural killer cell
NKT cell: natural killer T cell
nSMase: neutral sphyngomyelinase
PAFs: para-cancer fibroblasts
PBX3: pre-B-cell leukemia transcription factor 3
PI3K: phosphoinositide-3-kinase
PIGR: polymeric immunoglobulin receptor
PLD2: phospholipase D2
PS: phosphatidylserine
PSC: primary sclerosing cholangitis
SNARE: soluble N-ethylmaleimide-sensitive factor attachment protein receptor
TAMPs: tumor-associated microparticles
VEGF: vascular endothelial growth factor
VNN1: pantetheinase
VTDB: vitamin-D binding protein.
